# Relationships between plasma biomarkers, neuroimaging markers and cognition in cerebral amyloid angiopathy

**DOI:** 10.1002/alz70856_102306

**Published:** 2025-12-24

**Authors:** Ryan T Muir, Andrew E. Beaudin, Cheryl R. McCreary, Myrlene Gee, Krista Nelles, Nikita Nukala, Janina Valencia, Sophie K Stukas, Jennifer G Cooper, Kristopher M. Kirmess, Sandra E. Black, Michael D Hill, Cheryl L Wellington, Richard Camicioli, Eric E. Smith

**Affiliations:** ^1^ University of Toronto, Toronto, ON, Canada; ^2^ Sunnybrook Research Institute, Toronto, ON, Canada; ^3^ Hotchkiss Brain Institute, Calgary, AB, Canada; ^4^ University of Calgary, Calgary, AB, Canada; ^5^ University of Alberta, Edmonton, AB, Canada; ^6^ University of British Columbia, Vancouver, BC, Canada; ^7^ C2N Diagnostics, LLC, Saint Louis, MO, USA; ^8^ Division of Neurology, Department of Medicine, University of Toronto, Toronto, ON, Canada; ^9^ Dr. Sandra E. Black Centre for Brain Resilience and Recovery, LC Campbell Cognitive Neurology, Hurvitz Brain Sciences Program, Sunnybrook Research Institute, University of Toronto, Toronto, ON, Canada; ^10^ LC Campbell Cognitive Neurology Research Unit, Sunnybrook Research Institute, University of Toronto, Toronto, ON, Canada; ^11^ Sunnybrook Research Institute, University of Toronto, Toronto, ON, Canada; ^12^ Department of Clinical Neurosciences, University of Calgary, Calgary, AB, Canada; ^13^ Hotchkiss Brain Institute, University of Calgary, Calgary, AB, Canada

## Abstract

**Background:**

This study examined whether plasma Aβ_42/40,_ phosphorylated‐tau (*p*‐tau), neurofilament light chain (NfL) and glial fibrillary acidic protein (GFAP) may relate to cognitive function and other neuroimaging biomarkers in cerebral amyloid angiopathy.

**Method:**

This is a cross‐sectional analysis of a prospective cohort study of participants with CAA who had plasma collected, underwent magnetic resonance imaging (MRI) and cognitive evaluation. Plasma Aβ was quantified independently through Simoa and immunoprecipitation‐mass spectrometry (IP‐MS), while *p*‐tau181, NfL and GFAP were quantified through Simoa.

**Result:**

There were 45 participants with probable CAA (Boston criteria v2.0) eligible for analysis. In multivariable linear regression, adjusting for age, sex, and years of education, higher NfL levels were associated with lower Montreal Cognitive Assessment (MoCA) total score (β=‐1.85, *p* = 0.02) while lower Aβ_42/40_ was associated with lower speed of processing both with IP‐MS (β =0.63, *p* = 0.003) and Simoa (β =0.38, *p* = 0.02) methodology. After adjustment for age and sex, higher white matter hyperintensity (WMH) volume was associated with lower Aβ_42/40_ (IP‐MS), β =‐0.48, *p* = 0.009 and higher *p*‐tau181, β =0.35, *p* = 0.04, but not NfL or GFAP. Higher concentrations of plasma GFAP were associated with lower total cortical volumes (β =‐0.40, *p* = 0.042). Furthermore, higher levels of NfL were associated with lower global cerebrovascular reactivity (β=‐0.46, *p* = 0.008) and higher ordinal CAA severity scale score (aOR = 2.25, 95% CI: 1.17, 4.36, *p* = 0.02).

**Conclusion:**

Lower plasma Aβ_42/40_ was associated with lower speed of processing function, while plasma NfL was associated with worse performance on a global measure of cognitive performance, lower cerebrovascular reactivity, and higher CAA severity score. Interestingly, higher *p*‐tau181 and lower Aβ_42/40_ (IP‐MS) was associated with higher WMH volumes. These findings suggest that cognitive impairment in patients with CAA may be associated with markers of progressive white matter damage rather than tau‐related cortical injury.

